# Supplementary material for: Financial relief policy and social distancing during the COVID-19 pandemic
Source: PLOS Glob Public Health. 2022 Jun 30;2(6):e0000663. doi: 10.1371/journal.pgph.0000663 (PMC10021646; doi:10.1371/journal.pgph.0000663)
Supplement: Attachment — Submitted filename: response.pdf [file pgph.0000663.s001.pdf]

Response to the Referees' Reports for *PLOS Global Public Health*: (PGPH-D-22-00258)

Financial relief policy and social distancing during the COVID-19 pandemic

We are grateful for your comments and suggestions. Your report raises a number of points that indeed should be further explored and clarified in order to improve the paper. In addition, we thank you for providing concrete advice on revising our paper. We summarize each of the points of your letter and discuss how we incorporated your comments and suggestions in the revised manuscript. Hereafter, all the stated page numbers correspond to the revised version of our paper.

Response to Referee 1 comments

Comment 1: *Although the paper examines an interesting research question, the authors fail to discuss why they run different regressions without controlling many other independent variables, potentially impacting mobility (social distancing). For example, during the COVID period, we know many other stringency policies are implemented by policymakers, potentially impacting social distancing. In other words, during the analysis period of the paper, many different countries implemented many control policies outside of financial relief policy. In this context, how can the paper say that the change in mobility is caused by financial relief policy without controlling the potentially effect of other policy implementations such as international travel controls, cancellation of public events, closing of public transportation, the closing of schools, closing of workplaces, restrictions on internal movements, etc. (please see Oxford COVID-19 Government Response Tracker database, Thomas et al., 2020). For example, the policymakers may have applied less stringent policies in the relevant period, and mobility may have increased due to this implementation.*

Our Response: The emergency declaration was nationwide so the control variable ED_{ct} indicating the emergency declaration is strongly informative of distancing policy over the period of analysis, and further indicates the most devastating period of the pandemic as well. Other policy variables during this period would be highly correlated with ED_{ct} and

thus would cause problems for the regression estimation. We have included a new paragraph and a new figure (Fig 2) on Page 5 to demonstrate the validity of our data:

"Fig 2 shows the periods of emergency declarations and CARES Act stimulus payments in our sample coverage. The nationwide emergency for COVID-19 was declared on March 13th, right after the WHO declared COVID-19 a pandemic on March 11th, 2020. However, some states declared an emergency in early March. The emergency status continued as the United States recorded 50,000 new cases of COVID-19 on July 2nd, the largest one-day spike since the pandemic's onset.[1] States postponed or reversed plans to reopen their economies in July. Thus, our sample period covers the most devastating pandemic period in the United States when the social distancing order was enforced. The payment period in our sample starts from April 17th, 2020. Therefore, our sample consists of balanced coverage of normal, emergency, and stimulus payment periods."

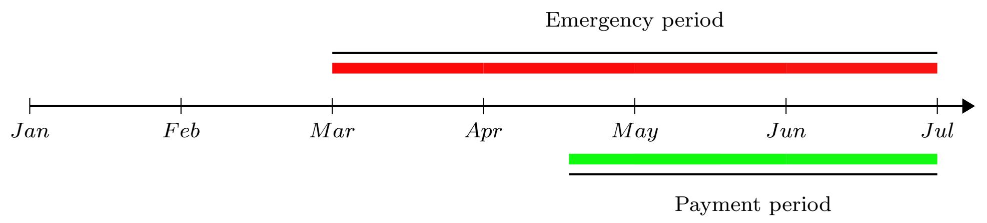

Figure 2: **Timeline of emergency declarations and CARES Act stimulus payments in our sample coverage in 2020.**

To address the comments on the effects of relief policy, we provide a detailed description of our regression model:

$$\begin{aligned} Y_{ct} = &\sum_{q \in Q} c_q \cdot D_q + \sum_{q \in Q} \alpha_q \cdot D_q \times D_t^{wknd} + \sum_{q \in Q} \beta_q \cdot D_q \times ED_{ct} \\ &+ \sum_{q \in Q} \gamma_q \cdot D_q \times ED_{ct} \times D_t^{wknd} + \sum_{q \in Q} \theta_q \cdot D_q \times ED_{ct} \times D_t^{pay} \\ &+ \sum_{q \in Q} \eta_q \cdot D_q \times ED_{ct} \times D_t^{pay} \times D_t^{wknd} + \zeta \cdot X_{ct} + \epsilon_{ct}. \end{aligned} \tag{1}$$

[1]See the timeline of COVID-19 developments in 2020 on the website of the American Journal of Managed Care https://www.ajmc.com/view/a-timeline-of-covid19-developments-in-2020.

We also add the following paragraph on Page 5 and a new table on Page 6:

"Model (1) is sometimes referred to as the saturated regression which utilizes dummies and their interactions to disentangle various marginal effects. Table 1 summarizes the marginal effects on mobility estimated from model (1). Since our sample provides balanced coverage of different episodes over the period, the sample size is sufficient to obtain consistent estimates."

Table 1: Summary of marginal effects on mobility measures

Regressor	Marginal effects estimated
D_q	Average daily mobility of people in income quintile q in *normal period*
$D_q \times D_t^{wknd}$	Weekend extra mobility of people in income quintile q in *normal period*
$D_q \times ED_{ct}$	Daily extra mobility of people in income quintile q in *emergency period*
$D_q \times ED_{ct} \times D_t^{wknd}$	Weekend extra mobility of people in income quintile q in *emergency period*
$D_q \times ED_{ct} \times D_t^{pay}$	Daily extra mobility of people in income quintile q *after the first stimulus payment*
$D_q \times ED_{ct} \times D_t^{pay} \times D_t^{wknd}$	Weekend extra mobility of people in income quintile q *after the first stimulus payment*

Notes: D_q, $q = 1, \ldots, 5$, is the five income quintile dummies, D_t^{wknd} is the weekend dummy, ED_{ct} indicates the after state emergency declaration period in county c, and D_t^{pay} indicates the stimulus payment period.

Comment 2: *The author should express the difference between this study from the common literature (see Mendolia et. al., 2020).*

Our Response: We have added a paragraph to discuss the differences between our study from the common literature such as (Mendolia et al. [2021]). The revised paragraph on Page 2 is as follows:

"Many epidemiologists have sounded alerts regarding the COVID-19 infection spread. Early literature demonstrates the evidence of reduction in human mobility with significantly reducing virus growth rates (see Fang et al. [2020] and Shibamoto et al. [2022]). A growing literature studies the impact of the government-implemented policies on human behavior.

Mendolia et al. [2021] documents the stringent policies (e.g., restrictions on international travel, closures of schools and workplaces) have negative and statistically significant effects on human mobility. Other than the previous studies focusing on the government-imposed behavioral policies, our study further investigates the impact of financial relief policy on human mobility."

Comment 3: *The article must support the reliability of empirical findings by adding different control variables to the model. In other words, the paper needs to present a robustness check of their empirical findings.*

Our Response: We agree with the reviewer that a robust study is needed to support our findings. As pointed out in the response to Comment 1, it is problematic to include more policy variables in the regression due to correlation with the emergency declaration dummy ED_{ct}. Thus, we collected new policy control data, namely the closure period of restaurants, gyms, movie theaters, and bars in different states as an alternative measure. The business closure period can be regarded as a *micro* level social distancing policy measure, whereas the emergency declaration as a *macro* level measure. These results are contained in a new Robustness section, and reaffirm the findings in the previous model, demonstrating the robustness of our claim that the financial relief policy may in fact loosen social distancing practices.

Comment 4: *When results are interpreted, there is a need to draw out from the literature arguments to support or dispute the findings. There is an inadequate discussion of the results. As there is little discussion of the implications of the results, the reader finds it hard to interpret the results. In other words, it is necessary to identify the wider implications of this set of findings. In the absence of a satisfactory discussion, the reader is left very much in the dark as to the specifics of the implications of the results.*

Our Response: We have discussed (see the response in Comment 2) how our study is relatively new to the common literature which mostly focuses on government-implemented policies on human behavior. Our work focuses primarily on the effects of financial aid policy, for which not much information can be drawn from the current literature. Nevertheless, we have carefully reviewed the supporting evidence of our results from the literature and included the policy implications of our findings. The revised paragraph on Page 7 is as follows:

“Overall, in line with the findings in Weill et al. [2020], our regression results show that the adoption of social distancing habits following states’ emergency declarations was substantial and strongly differentiated by income level, but that stimulus payments worsened social distancing behavior, especially on weekends. These results are unsurprising given that the stimulus payment is distributed on Friday, just in time for recipients to go shopping on the weekend. The results are consistent with the work of Yang et al. [2021] that stimulus payment has a significant short-term effect of boosting spending. Furthermore, although social distancing habits generally tended to lapse more for lower income quintiles, when looking specifically at retail activity it was in fact the middle income quintiles that showed the greatest increase. This can be explained by the result in Coibon et al. [2020], with the lowest income individuals using their stimulus checks to pay down debt and the highest income individuals saving theirs, leaving the middle income individuals most likely to actually spend the stimulus payment at retail. The findings has important policy implications for governments when the stimulus payment can be disbursed after weekends rather than before weekends to reduce the human mobility.”

Comment 5: *A possible ‘limitation’ should be stressed is that the USA may differ from other countries. If other researchers try to replicate this research, results may vary due to different countries. The Reviewer believes that it is important to stress this point.*

Our Response: We have pointed out the possible variation across countries and stated that this could be a future research topic. The revised paragraph on Page 12 is as follows:

“We would also like to emphasize that changes in human mobility in response to monetary policy can vary across countries that adopted different financial relief measures (e.g., tax deferrals and public guarantees to direct grants and coupons). It is interesting to compare the effects of different financial relief measures across countries during the COVID-19 pandemic. This could be explored in further research.”

Response to Referee 2 comments

***Comment 1**: Though paper tries to establish a negative relationship between economic stimulus and social distancing, however it is difficult to conclude whether economic support actually increased the social distancing at least for two reasons. First, it might just be natural to go for shopping on the weekend. We have this trend from centuries. Second, individuals might have not gone for those trips in the anticipation of economic support for which otherwise they might have gone. This information is subjective and not shown in the data. Authors may further recognise this as a shortcoming in the paper.*

Our Response: We would like to point out the that variable D_t^{wknd} and its interactions capture the differences between weekday and weekend mobility in the normal, emergency, and payment periods. We have provided a new descriptive paragraph and a table on Pages 5 and 6 as below:

$$\begin{aligned} Y_{ct} =& \sum_{q\in Q} c_q \cdot D_q + \sum_{q\in Q} \alpha_q \cdot D_q \times D_t^{wknd} + \sum_{q\in Q} \beta_q \cdot D_q \times ED_{ct} \\ &+ \sum_{q\in Q} \gamma_q \cdot D_q \times ED_{ct} \times D_t^{wknd} + \sum_{q\in Q} \theta_q \cdot D_q \times ED_{ct} \times D_t^{pay} \\ &+ \sum_{q\in Q} \eta_q \cdot D_q \times ED_{ct} \times D_t^{pay} \times D_t^{wknd} + \zeta \cdot X_{ct} + \epsilon_{ct}. \end{aligned} \tag{1}$$

"Model (1) is sometimes referred to the saturated regression which utilizes dummies and their interactions to disentangle various marginal effects. Table 1 summarizes the marginal effects on mobility estimated from model (1). Since our sample provides balanced coverage of different episodes over the period, the sample size is sufficient to obtain consistent estimates."

Table 1: Summary of marginal effects on mobility measures

Regressor	Marginal effects estimated
D_q	Average daily mobility of people in income quintile q in *normal period*
$D_q \times D_t^{wknd}$	Weekend extra mobility of people in income quintile q in *normal period*
$D_q \times ED_{ct}$	Daily extra mobility of people in income quintile q in *emergency period*
$D_q \times ED_{ct} \times D_t^{wknd}$	Weekend extra mobility of people in income quintile q in *emergency period*
$D_q \times ED_{ct} \times D_t^{pay}$	Daily extra mobility of people in income quintile q *after the first stimulus payment*
$D_q \times ED_{ct} \times D_t^{pay} \times D_t^{wknd}$	Weekend extra mobility of people in income quintile q *after the first stimulus payment*

Notes: D_q, $q = 1, \ldots, 5$, is the five income quintile dummies, D_t^{wknd} is the weekend dummy, ED_{ct} indicates the after state emergency declaration period in county c, and D_t^{pay} indicates the stimulus payment period.

References

Silvia Mendolia, Olena Stavrunova, and Oleg Yerokhin. Determinants of the community mobility during the covid-19 epidemic: The role of government regulations and information. *Journal of Economic Behavior & Organization*, 184:199–231, 2021.

Hanming Fang, Long Wang, and Yang Yang. Human mobility restrictions and the spread of the novel coronavirus (2019-nCoV) in china. *Journal of Public Economics*, 191:104272, 2020.

Masahiko Shibamoto, Shoka Hayaki, and Yoshitaka Ogisu. Covid-19 infection spread and human mobility. *Journal of the Japanese and international economies*, 64:101195, 2022.

Joakim A. Weill, Matthieu Stigler, Olivier Deschenes, and Michael R. Springborn. Social distancing responses to COVID-19 emergency declarations strongly differentiated by income. *Proceedings of the National Academy of Sciences*, 117(33):19658–19660, 2020.

Zhiqing Yang, Youngjun Choe, and Matthew Martell. COVID-19 economic policy effects on

consumer spending and foot traffic in the us. *Journal of Safety Science and Resilience*, 2 (4):230–237, 2021.

Olivier Coibon, Yuriy Gorodnichenko, and Michael Weber. How did U.S. consumers use their stimulus payments? *NBER Working Paper*, 2020.
